# The most accurate autonomic function test: the medical history

**DOI:** 10.1007/s10286-017-0449-z

**Published:** 2017-07-14

**Authors:** Janice L. Gilden

**Affiliations:** 1 James A. Lovell Federal Health Care Center, 3001 Greenbay Road, #111E, North Chicago, IL 60064 USA; 2 Section of Diabetes and Endocrinology, Department of Medicine, The Chicago Medical School at Rosalind Franklin University of Medicine and Science, 3333 Green Bay Rd, North Chicago, IL USA

“Data! Data! Data! I can’t make bricks without clay” stated the famous detective Sherlock Holmes, a fictional character of the British writer, Sir Arthur Conan Doyle. It is true that data are needed to draw a proper conclusion. In patients with autonomic dysfunction, every symptom is a “clue” or “piece of data”, that must be precisely evaluated and interpreted by a well-trained eye. This is required in order to arrive at the correct solution of the case. Thus, the second paper of the series on Autonomic Function Tests by Goldstein and Cheshire, published in this issue of *Clinical Autonomic Research*, highlights the importance of the medical history, the most accurate autonomic function test [1, 2]. They also emphasize the need to rule out other disorders and to be certain that the patient truly has autonomic dysfunction.

Autonomic medicine is a relatively new and rapidly evolving field, and it has been under-recognized by the medical community. Therefore, it is crucial to educate multidisciplinary providers, as well as to support further research in this area. Hence, this paper is quite timely. The authors summarize the relevant and important steps required to perform a comprehensive autonomic medical history, which—let me reiterate—is the most important autonomic test [2]. It is crucial to query the patient’s account of daily activities and the chronology relating to the autonomic nervous system (ANS), and then to recognize that these symptoms may also fluctuate from day to day. The medical history should focus upon various aspects of autonomic disorders with regard to: *Chief Complaint* (the description by the patient for the reason of this evaluation); *History of the Present Illness* (history of the condition with chronology and patterns of autonomic symptoms, prior evaluations, including review of medical records, family observations); *Past Medical History* (past medical issues, including toxic exposures, chemotherapy, or Lyme disease); *Family History* (medical conditions of family members, especially those related to the patient’s current medical problems, such as neurologic, cardiac, endocrine, or genetic disorders (e.g., inherited synucleinopathies), and hyperflexibility syndromes, which are sometimes present in patients diagnosed with postural tachycardia syndrome (POTS); *Current Medications, Dietary Supplements, and Herbal Remedies; Personal and Social History* (living situation, daily functioning, disability, smoking, alcohol, substance abuse, past traumas and physical abuse, car accidents, stresses); and *Review of Systems* (with focus on specific components of the ANS). Goldstein and Cheshire also include less commonly recognized symptoms, such as: “water bottle sign”, “coat hanger pain”, shifting weight back and forth while standing, leg fidgeting while seated, physical deconditioning, and pretzel legs [2]. Olfactory dysfunction, visual hallucinations (a feature of dementia with Lewy bodies) and dream enactment behavior (rapid eye movement behavior disorder), visceral hypersensitivity or “*somatic hypervigilance*”, as described by Khurana [3], may point to specific disorders. The authors also highlight the importance of having an appropriately trained and experienced clinician who is knowledgeable in asking the correct questions and interpreting the subjective answers, with the ability to discriminate between the autonomic functions and non-autonomic aspects of different forms of dysautonomia as well as an awareness that patients may have other non-associated issues and incorrect interpretations of the connection between these and the autonomic disorders, which are often influenced by educational, ethnic, cultural and language backgrounds, and social media.

Goldstein and Cheshire also emphasize that it is crucial to determine the type of autonomic dysfunction (specific to the sympathetic noradrenergic, sympathetic cholinergic, and parasympathetic nervous systems) [2]. One must also realize that even within the subcategories of ANS, the symptoms can still be quite heterogenous.

## Can questionnaires assist in obtaining the medical history?

Several questionnaires have been developed to assist in the evaluation of the individual with autonomic symptoms. However, few have proven universally applicable to all autonomic disorders, and some lack discriminatory value. The Composite Autonomic Symptom Score (COMPASS), with 31 questions, was developed by Low and colleagues to standardize evaluation of autonomic symptoms; it contains six domains: orthostatic intolerance, vasomotor, secretomotor, pupillomotor, bladder, and gastrointestinal [4]. However, as pointed out by Goldstein and Cheshire, gender-specific erectile dysfunction, the sequence or chronology of symptoms, as well as the syndromic nature of certain dysautonomias were not included. In addition, the COMPASS may not be applicable to individuals with POTS and related syndromes [2]. Using a symptom assessment tool, Robinson-Papp and colleagues recently evaluated 97 adults in an urban outpatient neurology clinic of an academic medical center and compared the results of their evaluation to those of a formal neurologic exam and standard autonomic function tests, as well as the Carlson Co-Morbidity Index (a measure of chronic disease burden) [5]. The questionnaire of symptoms proved to lack specificity, since the symptoms were more highly associated with the burden of chronic disease.

## History of other long-term consequences and risks

Although not generally recognized, other long-term consequences exist and include chronic fatigue, altered memory and concentration, panic/anxiety, suicidal ideation (recently described in patients with POTS), and syncope with falls resulting in bodily injuries [6]. When operating motor vehicles, there are risks of losing control, mental fatigue, confusion, and syncope resulting in collisions, similar to individuals with diabetes mellitus and hypoglycemia-related neuroglycopenia, as well as disrupted cognitive-motor functioning [7].

## Can the medical history be automated?

As the population ages, risk factors for the development of these autonomic disorders will also increase. Although the current electronic age places an emphasis on time constraints with automation of medical visits and the use of pre-determined questionnaires, accompanied by decreasing value on cognitive skills, there is still no substitute for a skilled and trained clinician who can carefully decipher the medical history of a patient with an autonomic disorder.

Therefore, it is crucial to consider every “clue” or “piece of data” and develop proficiency in obtaining a careful and precise medical history, the most important autonomic function test.
